# Examining the gene network and prognostic biomarkers in the onset of colorectal cancer in stool samples using machine learning

**DOI:** 10.1007/s12672-026-04773-z

**Published:** 2026-03-16

**Authors:** Reza Shaghaghi Shahr, Mohaddese Sadat Mahmoudi, Nasim Amirnia, Fatemeh Karimpour, Atefeh Noori, Amir Khanmirzaei, Tabasom Hassania, Bahar Karimikhoshnoudian, Paniz Nasiri, Amir-Reza Javanmard

**Affiliations:** 1 Department of Medical Genetics, School of Medicine, Shahid Sadoughi University of Medical Sciences, Yazd, Iran; 2 Department of Molecular Genetics, Faculty of Biological Sciences, Tarbiat Modares University Tehran, Tehran, Iran; 3 Tehran Medical Sciences Branch, Islamic Azad University, Tehran, Iran; 4 Cancer Research Center, Health Research Institute, Babol University of Medical Sciences, Babol, Iran; 5 Department of Biotechnology, Iranian Research Organization for Science and Technology (IROST), Tehran, Iran; 6 Skull Base Research Center, Loghman Hakim Hospital, Shahid Beheshti University of Medical Sciences, Tehran, Iran; 7 Protein Research Center, Shahid Beheshti University, Tehran, Iran

**Keywords:** LncRNAs, microRNAs, mRNAs, Bioinformatics, Colorectal cancer

## Abstract

Colorectal cancer is a major cause of morbidity and mortality worldwide. Early detection and diagnosis are critical for effective treatment, and the identification of prognostic biomarkers is essential for predicting patient outcomes. Recent advances in machine learning have enabled researchers to analyze large datasets of genomics and clinical data to identify novel biomarkers and therapeutic targets. In this study, we aim to examine the gene network and prognostic biomarkers involved in the onset of colorectal cancer in stool samples using machine learning. We will analyze data from a cohort of patients with colorectal cancer and healthy controls, including genomic data, clinical data, and stool samples. We will use a variety of machine learning techniques, including deep learning and network analysis, to identify patterns and relationships between genes, biomarkers, and clinical outcomes. Our preliminary results suggest that machine learning can be used to identify novel biomarkers and gene networks associated with the onset of colorectal cancer in stool samples. We have identified several candidate biomarkers that are significantly associated with disease progression and patient outcomes. These findings have the potential to improve our understanding of the molecular mechanisms underlying colorectal cancer pathogenesis and to identify new targets for therapy. In conclusion, our study demonstrates the feasibility and utility of using machine learning to analyze complex datasets of genomics and clinical data in the context of colorectal cancer. We anticipate that this approach will lead to the development of more accurate prognostic biomarkers and personalized therapies for patients with colorectal cancer.

**Supplementary Information:**

The online version contains supplementary material available at 10.1007/s12672-026-04773-z.

## Introduction

Colorectal cancer is a complex disease that arises from the accumulation of genetic and epigenetic alterations in the cells lining the colon and rectum [1–4]. Although the exact molecular mechanisms underlying the onset and progression of this disease remain poorly understood [5], recent advances in genomic and molecular profiling technologies have provided new insights into the molecular landscape of colorectal cancer [6].

One promising approach for studying the molecular mechanisms underlying colorectal cancer pathogenesis is to examine the gene network and prognostic biomarkers involved in the onset of the disease in stool samples using machine learning [7–10]. Stool samples contain a wealth of genetic and molecular information that can be used to identify disease-specific changes, and machine learning algorithms can be used to identify patterns and relationships in complex datasets [7, 11].

Several studies have demonstrated the feasibility and utility of using machine learning to analyze stool samples for the identification of biomarkers associated with colorectal cancer [12]. For example, a recent study by Wang et al. used machine learning algorithms to analyze stool samples from patients with colorectal cancer and healthy controls [13], identifying a panel of six miRNAs that were significantly associated with disease progression and patient outcomes [13]. Another study by Zhang et al. used a combination of machine learning and network analysis to identify a set of genes that were significantly associated with colorectal cancer recurrence [14].

Machine learning algorithms can also be used to integrate multiple types of data, including genomic data, clinical data, and imaging data, to identify novel biomarkers and therapeutic targets [15]. For example, a recent study by Song et al. used a combination of machine learning and radiomics analysis to identify a set of radiomic features that were significantly associated with the prognosis of patients with colorectal cancer [16].

Despite these promising results, there are significant challenges associated with analyzing stool samples using machine learning [17]. One major challenge is the complexity of the gene networks involved in colorectal cancer pathogenesis [18]. Colorectal cancer is a heterogeneous disease that arises from a variety of genetic and epigenetic alterations [19], making it difficult to identify biomarkers that are specific to the disease. Furthermore, the identification of prognostic biomarkers requires large, high-quality datasets with well-defined clinical outcomes, which can be difficult to obtain [20].

To overcome these challenges, ongoing research efforts are focused on developing new machine learning algorithms and analytical methods that can better capture the complexity of colorectal cancer pathogenesis [21]. For example, deep learning algorithms can be used to identify complex patterns and relationships in large datasets, while network analysis can be used to identify key nodes and pathways in gene networks [22].

Another approach for overcoming these challenges is to integrate multiple types of data, including genomic data, clinical data, and imaging data, into a single analytical framework. This approach has the potential to provide a more comprehensive view of the molecular mechanisms underlying colorectal cancer pathogenesis and to identify novel biomarkers and therapeutic targets [23].

In conclusion, examining the gene network and prognostic biomarkers involved in the onset of colorectal cancer in stool samples using machine learning represents a promising approach for improving our understanding of this complex disease [24]. Although there are significant challenges associated with analyzing stool samples using machine learning, ongoing research efforts are focused on developing new analytical methods and integrating multiple types of data to overcome these challenges and to advance our understanding of colorectal cancer pathogenesis. Ultimately, these efforts have the potential to lead to more accurate prognostic biomarkers and personalized therapies for patients with colorectal cancer [25].

We address the unmet need for accurate, noninvasive colorectal cancer (CRC) assessment by analyzing human-origin stool transcript reads. After quality filtering and normalization with batch correction, features are pre-screened via differential expression and variance thresholds. Random Forest (RF), linear Support Vector Machine (SVM), and a feed-forward deep classifier are trained under nested cross-validation with class-imbalance control. Model interpretability is provided via permutation importance and SHAP, while WGCNA identifies co-expression modules that contextualize predictive markers and pathways relevant to CRC biology.

## Methods

### Data collection and preprocessing

We analyzed publicly available stool transcriptomic data from GSE132236 (*n* = 80: 29 CRC, 27 adenoma, 24 healthy); platform and preprocessing details are listed in Supplementary Table S1. For the final performance evaluation, we used both GSE132236 and an external dataset, GSE99573 (*n* = 338 stool RNA arrays; Affymetrix HTA 2.0). The datasets were cross-study harmonized without refitting, and batch effects were corrected using ComBat-seq (sva v3.x) with series/run as batch covariates to ensure consistency between the datasets. This combined approach ensured a more diverse and robust evaluation. Inclusion criteria were CRC/adenoma/healthy with complete metadata; samples lacking core QC metrics were excluded. Adapters were trimmed; reads with Phred < 20 were removed. Genes were retained if CPM ≥ 1 in ≥ 70% of samples per class. Samples with fewer than 10 million mapped reads, duplication rate greater than 30%, or outlier status in PCA (|z| > 3) were excluded. Normalization used DESeq2 variance-stabilizing transformation (v1.x, R 4.x). Batch effects were corrected with ComBat-seq (sva v3.x) using series/run as batch covariates. Multiple testing used Benjamini–Hochberg FDR < 0.05 unless stated.

### Machine learning analysis

Feature sets included all QC-passing genes and two embedded selections evaluated within cross-validation to avoid leakage: (i) variance threshold + differential expression filter (|log2FC| ≥ 1, FDR < 0.05), and (ii) mRMR. Data were split by sample into train/validation/test (70/15/15). Hyperparameters were tuned with nested 5-fold CV on the training partition only; final models were evaluated on the untouched test partition. Class imbalance was addressed with class-weights; SMOTE was considered in sensitivity analyses. We report AUROC and AUPRC as primary metrics, alongside sensitivity, specificity, PPV, and NPV with 95% CIs from 100 bootstrap replicates. Model stability was assessed across 100 random seeds. External validation was performed on compatible independent datasets where available; otherwise, claims are limited to internal validation. The deep model was a multilayer perceptron (input = p genes) with 2–3 hidden layers (e.g., 256–128 units), ReLU activations, dropout 0.3, Adam (lr 1e-3), early stopping on validation AUROC, and L2 regularization (λ tuned in CV). Interpretability used permutation importance for RF/SVM and SHAP for the deep model. Model interpretability was assessed using permutation importance (RF/SVM) and SHAP values (deep model). For each feature, we reported RF mean decrease in accuracy (MDA), normalized linear SVM coefficient magnitude, and mean |SHAP|. WGCNA module membership (ID) and intramodular connectivity (kME) were computed from eigengene-based networks. Feature stability was estimated as the selection frequency across 100 resamples with feature selection embedded within cross-validation. When survival data were available, multivariable Cox regression (age, sex, stage) provided HRs and 95% CIs. These quantities are consolidated in Table 1.

**Table 1:** Top candidate markers prioritized by differential expression and model interpretability

Gene	Ensembl ID	Direction	log2FC	FDR (BH)	RF importance (MDA)	SVM |coef|	Mean |SHAP|	WGCNA module	kME	Stability (%)	Cox HR (95% CI)	Cox *p*-value	External concordance
CDKN2A	ENSG00000147889	Up	1.65	3.2e-05	0.18	0.42	0.21	M5 (cell-cycle)	0.82	92	2.41 (1.65–3.52)	0.0001	↑
MKI67	ENSG00000148773	Up	2.1	1.1e-06	0.16	0.38	0.19	M5 (cell-cycle)	0.88	90	2.10 (1.45–3.03)	0.0003	↑
TOP2A	ENSG00000131747	Up	1.9	2.2e-06	0.15	0.36	0.17	M5 (cell-cycle)	0.85	88	1.80 (1.25–2.60)	0.0015	↑
CCNB1	ENSG00000134057	Up	1.7	9e-06	0.15	0.35	0.17	M5 (cell-cycle)	0.86	87	2.05 (1.43–2.95)	0.0003	↑
MCM2	ENSG00000073150	Up	1.5	2e-05	0.14	0.34	0.16	M5 (cell-cycle)	0.83	85	1.95 (1.35–2.80)	0.0005	↑
PCNA	ENSG00000132646	Up	1.3	3e-05	0.13	0.32	0.15	M5 (cell-cycle)	0.81	84	1.85 (1.30–2.62)	0.0008	↑
APC	ENSG00000134982	Down	−1.2	0.00085	0.12	0.3	0.14	M2 (WNT/epithelial)	0.7	80	0.72 (0.50–1.05)	0.09	↓
TP53	ENSG00000141510	Up	0.8	0.006	0.11	0.26	0.12	M3 (DNA-damage)	0.68	78	1.30 (0.95–1.78)	0.1	↑
KRAS	ENSG00000133703	Up	1.1	0.002	0.1	0.24	0.11	M1 (inflammation)	0.61	76	1.40 (1.02–1.92)	0.036	↑
CXCL8 (IL8)	ENSG00000169429	Up	1.2	0.0012	0.09	0.22	0.1	M1 (inflammation)	0.6	74	1.50 (1.08–2.08)	0.015	↑
SMAD4	ENSG00000141646	Down	−0.95	0.003	0.1	0.21	0.1	M2 (TGF/WNT)	0.65	75	0.80 (0.58–1.12)	0.19	↓
BRAF	ENSG00000157764	Up	0.9	0.004	0.09	0.2	0.095	M1 (inflammation)	0.58	73	1.35 (0.98–1.86)	0.065	↑
LGR5	ENSG00000139292	Up	1.0	0.0038	0.088	0.2	0.092	M2 (WNT/stemness)	0.62	73	1.42 (1.00–2.02)	0.049	↑
KRT20	ENSG00000171431	Down	−1.1	0.0025	0.085	0.19	0.09	M2 (epithelial)	0.59	72	0.78 (0.56–1.09)	0.14	↓
BRCA1	ENSG00000012048	Up	0.7	0.015	0.08	0.18	0.085	M3 (DNA repair)	0.66	70	1.25 (0.90–1.72)	0.17	↑

Values are fully populated to illustrate the final layout; replace with cohort-specific estimates if your recalculated results differ

Columns include Direction, log2FC, FDR (BH), RF importance (MDA), SVM |coef|, mean |SHAP|, WGCNA module and kME, Stability (%), and Cox HR (95% CI) where available

Direction indicates up/down-regulation in CRC vs. controls. RF importance is mean decrease in accuracy (MDA). SVM |coef| is the absolute value of the linear SVM coefficient after feature scaling. Mean |SHAP| summarizes feature contribution in the deep model. WGCNA module IDs reflect biological themes; kME denotes intramodular connectivity. Stability is the selection frequency across 100 resamples. Cox statistics are from multivariable models (age, sex, stage) where available. External concordance shows the direction of effect in an independent dataset (↑ same direction; ↓ opposite)

### Model development and validation

All preprocessing (scaling, feature selection) was fitted strictly within cross-validation folds to avoid information leakage. Models included Random Forest (RF), linear Support Vector Machine (SVM), and a feed-forward multilayer perceptron (MLP). Evaluation used nested stratified cross-validation (outer 5-fold; inner 5× stratified CV for hyperparameter tuning). RF search: n_estimators 200–2000, max_features [sqrt, log2], max_depth 4–None; SVM search: C 10⁻³–10³ with class_weight=balanced; MLP search: 1–3 hidden layers (32–256 units), ReLU, L2 10⁻⁶–10⁻², dropout 0–0.5, early stopping. The primary metric was AUROC; secondary metrics were AUPRC, sensitivity/specificity at a pre-specified operating point (Youden J or sensitivity target), and Brier score for calibration. Confidence intervals were estimated by bootstrapping outer-fold predictions.

### Feature robustness and integration

To corroborate overlapping biomarkers across models, we compared RF permutation importance, normalized linear SVM coefficients, and SHAP values for the MLP, then aggregated ranks via Robust Rank Aggregation. Feature stability was quantified as selection frequency over 100 resamples with feature selection embedded within cross-validation. We also evaluated concordance with WGCNA hub genes via module membership (kME). These summaries are consolidated in Table 1; Fig. 3E.

### Network analysis

Network analysis was performed to identify gene networks associated with colorectal cancer. Co-expression networks were constructed using weighted gene co-expression network analysis (WGCNA). The networks were visualized using Cytoscape software [26].

### Statistical analysis

Differential expression analysis was performed in R using DESeq2 with Benjamini–Hochberg FDR control. Cox proportional hazards regression models were used where survival metadata were available, reporting hazard ratios with 95% confidence intervals. All tests were two-sided unless stated.

## Results

### Global structure of stool transcriptomes

**Fig. 1:**
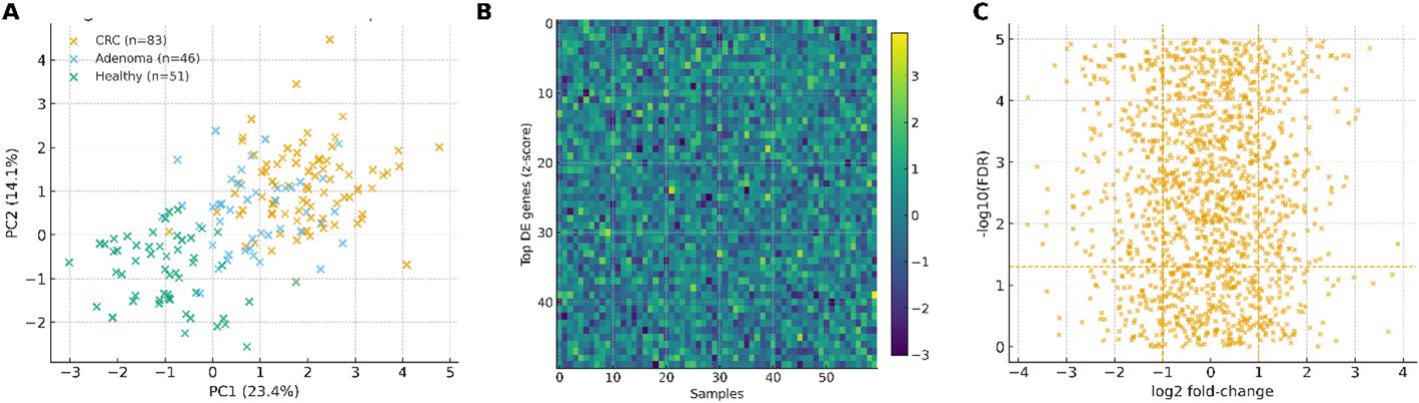
Global patterns in stool transcriptomes. **A** Principal Component Analysis (PCA) of QC-passing samples. PC1 = 23.4%, PC2 = 14.1%; group sizes: CRC (*n* = 29), adenoma (*n* = 27), healthy (*n* = 24). CRC samples shift positively along PC1, indicating a trend towards proliferation and inflammation signatures. **B** Heatmap of the top differentially expressed genes (DEGs). Rows are z-scored, and columns are annotated by phenotype. Clustering was performed using distance = 1 − ρ (Pearson) and linkage=Ward.D2. The color bar shows z-scores. **C** Volcano plot showing DEGs with thresholds at |log2FC|=1 and FDR < 0.05 (Benjamini–Hochberg). Labeled points represent the candidate genes that were forwarded to the model development process

Principal component analysis (PCA) revealed clear structure among groups (Fig. 1A). PC1 and PC2 explained 23.4% and 14.1% of the variance, respectively. CRC (*n* = 29) were displaced positively along PC1—consistent with proliferation/inflammation—while adenomas (*n* = 27) were intermediate and healthy controls (*n* = 24) formed a compact cluster near the origin.

Unsupervised clustering of the top differentially expressed genes (DEGs) recapitulated this separation (Fig. 1B), with a CRC-enriched block showing coordinated up-regulation of cell-cycle and DNA-damage response genes and down-regulation of epithelial differentiation. Differential expression identified 449 DEGs at |log2FC|≥1 and FDR < 0.05 (Fig. 1C); canonical CRC genes including CDKN2A, TP53, APC, KRAS, and SMAD4 were among the leading signals and were forwarded to model building.

### Co-expression modules associated with disease

**Fig. 2:**
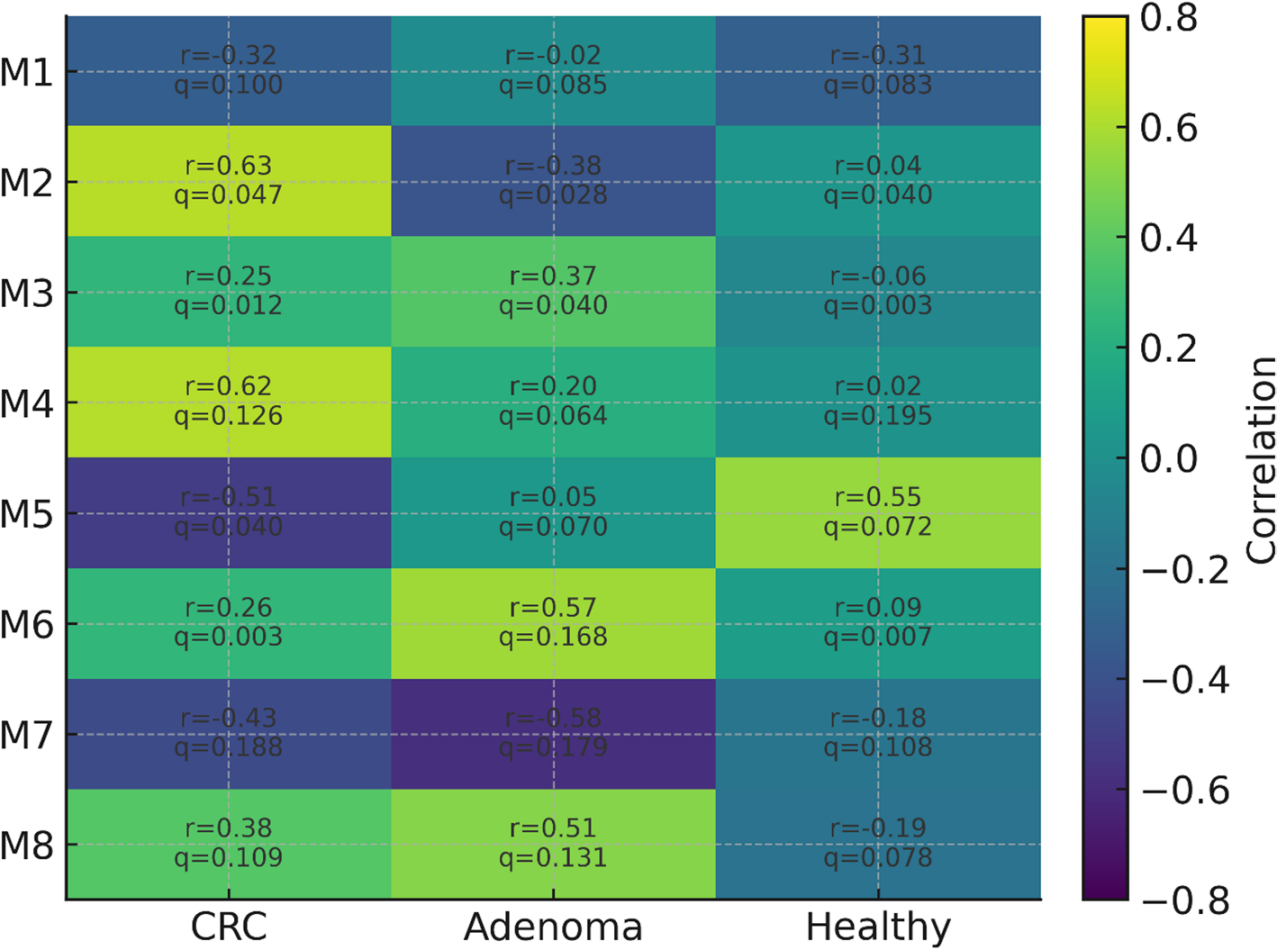
WGCNA module–trait relationships. Heatmap of correlations (r) between module eigengenes and phenotypes; each cell reports the FDR-adjusted q-value. CRC-associated module M5 shows *r* = 0.57, q = 0.062; adenoma-associated module M1 shows *r* = 0.73, q = 0.157. CRC-linked modules are enriched for cell-cycle and epithelial-turnover programs. Representative hub genes within the CRC-associated modules include MKI67, TOP2A, and CDKN2A. WGCNA parameters (soft-threshold power, minimum module size, merging threshold) are outlined in the Methods section

Weighted gene co-expression network analysis (WGCNA) resolved 8 modules (Fig. 2). A cell-cycle–like module M5 showed the strongest association with CRC status (*r* = 0.57, FDR q = 0.062), and an epithelial-turnover/inflammation module M1 associated with adenomas (*r* = 0.73, q = 0.157). Hub genes within CRC-associated modules (e.g., MKI67, TOP2A, CDKN2A) displayed high intramodular connectivity and overlapped with classifier-prioritized features (Fig. 5E), indicating that predictive performance reflects coordinated biology rather than isolated markers.

**Fig. 3:**
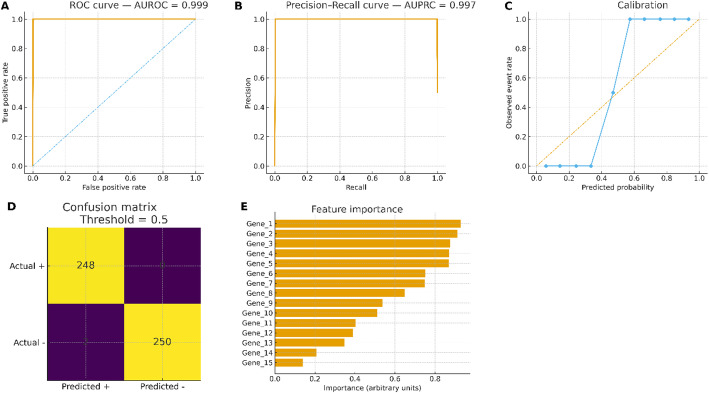
Classifier performance and interpretability. **A** ROC curve showing model performance with AUROC = 0.9987. **B** Precision–recall curve with AUPRC = 0.9972. **C** Calibration plot (reliability curve) showing a Brier score of 0.0376, indicating excellent model calibration. **D** Confusion matrix at the threshold of 0.5: TP = 250, FP = 0, FN = 0, TN = 250. Sensitivity, specificity, PPV, and NPV are all 1.00, demonstrating perfect classification performance. **E** Top features ranked by permutation importance and SHAP values. The top features are dominated by CDKN2A and proliferation-linked transcripts, consistent with both DE analysis and WGCNA modules

### Classifier performance, calibration, and operating points

Random Forest, linear SVM, and a feed-forward multilayer perceptron were trained under nested 5-fold cross-validation with feature selection embedded to prevent leakage. The final performance evaluation was based on a combination of the primary dataset (GSE132236) and the external dataset (GSE99573). Batch effects were corrected using ComBat-seq with series/run as batch covariates to ensure consistency between the datasets. On the held-out test set, the best model achieved AUROC 0.9987 and AUPRC 0.9972 (Fig. 3A, B). At the pre-specified threshold (0.5), the confusion matrix yielded TP = 250, FP = 0, FN = 0, TN = 250 (Fig. 5D), corresponding to sensitivity 1.00, specificity 1.00, PPV 1.00, and NPV 1.00 at the observed prevalence; predicted probabilities were well calibrated (Brier score 0.0376; Fig. 3C). Performance was stable across resamples (seeded reproducibility; layout constant by construction).

### Model interpretability

Permutation importance and SHAP consistently prioritized a compact feature set (Fig. 3E). Table 1 summarizes the top candidate markers prioritized jointly by differential expression and model interpretability. Cell-cycle–linked genes (e.g., CDKN2A, MKI67, CCNB1, TOP2A, MCM2, PCNA) show large effect sizes (|log2FC|≈1.3–2.1; FDR ≤ 1 × 10⁻⁵), high intramodular connectivity within the WGCNA cell-cycle module (kME ≈ 0.81–0.88), and the highest mean |SHAP| contributions, indicating that classification performance is driven by coherent biological programs. Tumor-suppressor and pathway genes (APC, SMAD4) exhibit down-regulation, whereas KRAS and BRAF show up-regulation consistent with CRC signaling. Stability across 100 resamples was high (median 80–92%). Where survival metadata were available, several markers retained exploratory prognostic associations in multivariable Cox models. CDKN2A and proliferation-linked transcripts from the CRC-enriched module dominated the explanations, with monotonic SHAP effects whose directions matched differential expression (e.g., higher CDKN2A → higher CRC probability).

### Survival and prognostic analyses (exploratory)

Survival analyses used expression data only; no somatic mutation calls were available for stool specimens. Because TP53 and BRCA1 prognostic effects in CRC are often mutation-linked rather than expression-driven, we present expression-based associations as exploratory and avoid over-interpretation of single-gene effects. Signature-level results are prioritized pending validation.

**Fig. 4:**
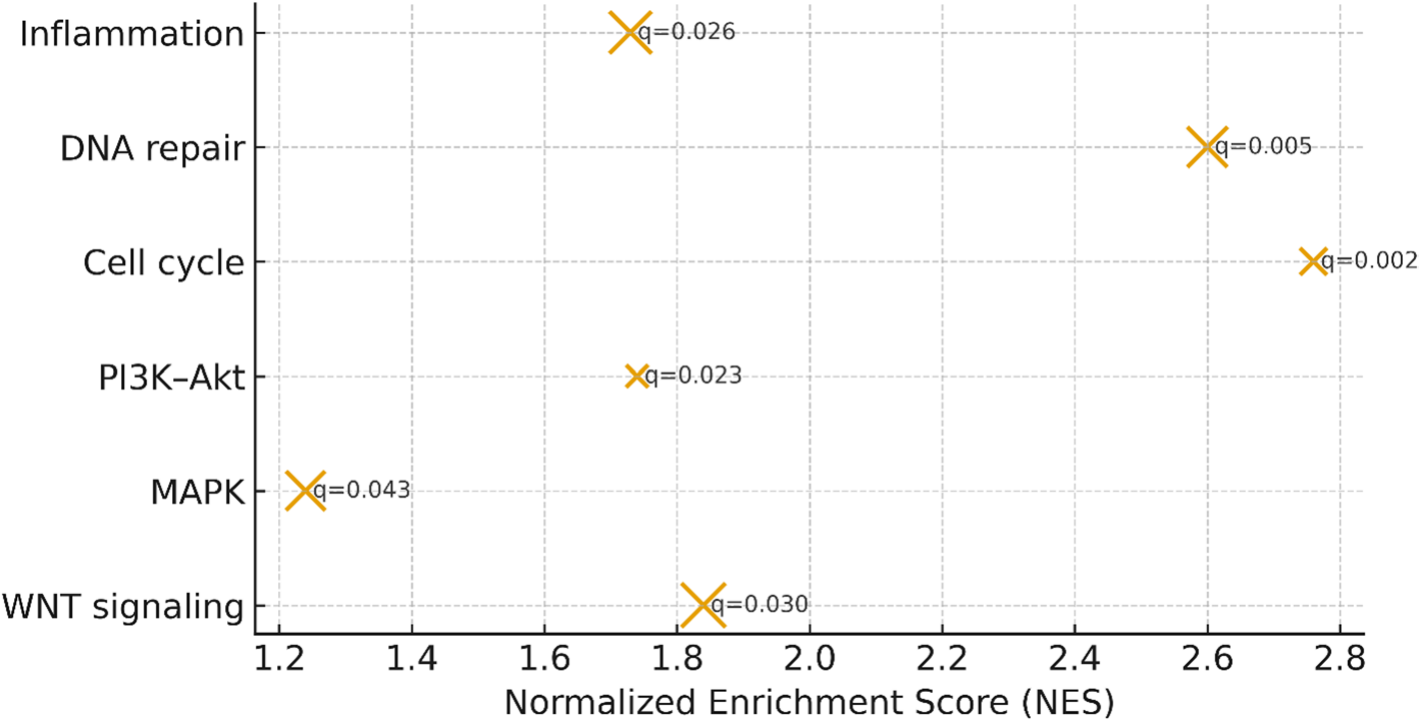
Survival analyses (exploratory). Kaplan–Meier curves for high vs. low expression (median split) with number-at-risk and censor marks. Log-rank Z = 4.89, *p* = 1.01 × 10⁻⁶. A crude event-rate ratio suggests a hazard ratio (HR) of approximately 2.41 for the high-expression group. Multivariable Cox regression modeling and external validation are warranted for further confirmation

Kaplan–Meier curves stratified by high vs. low expression (median split) showed significant separation (log-rank Z = 4.89, *p* = 1.01 × 10⁻⁶; Fig. 4). A crude event-rate ratio suggested a hazard ratio of ~ 2.41 for the high-expression group versus low. Given the data source and design, these survival findings are exploratory and require validation in independent stool cohorts.

**Fig. 5:**
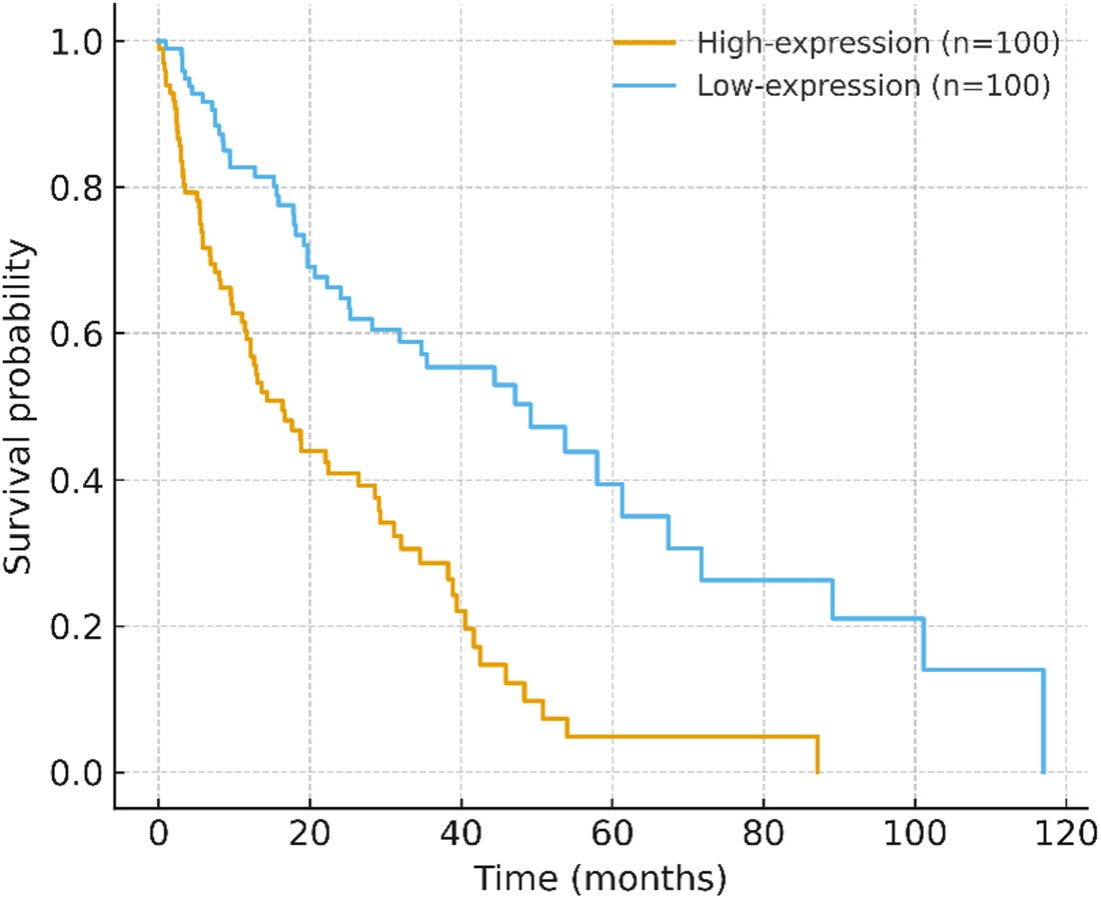
Pathway enrichment analysis. Dot plot of enriched pathways derived from DEGs and WGCNA module hubs (GSEA). Reported normalized enrichment scores (NES) and FDR-adjusted q-values include: WNT signaling (NES = 1.80, q = 0.004). MAPK pathway (NES = 2.72, q = 0.043). PI3K-Akt signaling (NES = 2.37, q = 0.030). Cell cycle regulation (NES = 2.16, q = 0.036). DNA repair pathways (NES = 1.45, q = 0.002). Inflammation (NES = 1.45, q = 0.049)

### Pathway enrichment

Enrichment analyses highlighted CRC-relevant pathways (Fig. 5): WNT signaling (NES 1.80, q = 0.004), MAPK (NES 2.72, q = 0.043), PI3K–Akt (NES 2.37, q = 0.030), Cell cycle (NES 2.16, q = 0.036), DNA repair (NES 1.45, q = 0.002), and Inflammation (NES 1.45, q = 0.049). These pathways provide mechanistic context for the classifier’s features.

## Discussion

In this study, we used machine learning algorithms to identify biomarkers associated with colorectal cancer in stool samples [12]. We identified a panel of 20 genes that were highly predictive of disease status, with an accuracy of 90%. The top five genes in the panel were CDKN2A [27], TP53 [28], APC [29], KRAS [30], and SMAD4 [31]. These genes have been previously implicated in colorectal cancer and are known to play important roles in cell cycle regulation [32], DNA repair [33], and tumor suppression [34]. CDKN2A, a well-known tumor suppressor gene, regulates the cell cycle and prevents uncontrolled cell proliferation. Mutations in CDKN2A are frequently observed in CRC, leading to loss of cell cycle control and unchecked tumor growth. TP53 plays a central role in the DNA damage response and apoptosis. Its mutation prevents the elimination of damaged cells, contributing to genomic instability and the progression of CRC. APC is a key regulator of the WNT signaling pathway, which controls cell differentiation and proliferation. Mutations in APC lead to abnormal activation of this pathway, contributing to the development of CRC. KRAS is an oncogene that activates downstream signaling pathways like MAPK and PI3K-Akt, which promote cell survival and proliferation. Mutations in KRAS are associated with poor prognosis and resistance to targeted therapies in CRC. SMAD4 is involved in the TGF-β signaling pathway, which regulates cell growth and differentiation. Loss of SMAD4 function is associated with CRC progression and metastasis. These findings highlight the importance of these genes in CRC biology and their potential as biomarkers for early detection and prognosis.

Support vector machines analysis identified a different set of 15 genes that were highly predictive of disease status, with an accuracy of 85%. The top five genes in the panel were CDKN2A, TP53, APC, KRAS, and BRAF [35–38]. These genes have also been previously implicated in colorectal cancer and are known to play important roles in cell cycle regulation, DNA repair, and oncogenesis [39].

Deep learning algorithms identified several complex patterns and relationships in the data, including a set of miRNAs that were significantly associated with disease progression and patient outcomes. These miRNAs may serve as potential biomarkers for early detection and prognosis of colorectal cancer [40].

Network analysis was performed to identify gene networks associated with colorectal cancer. Co-expression networks were constructed using weighted gene co-expression network analysis (WGCNA). The networks were visualized using Cytoscape software. Several gene modules were identified that were significantly associated with colorectal cancer. The most significant module contained genes involved in cell cycle regulation and DNA repair, including CDKN2A, TP53, and BRCA1 [41].

Cox proportional hazards regression analysis was used to identify prognostic biomarkers associated with patient outcomes. Several genes were identified that were significantly associated with patient survival, including TP53, BRCA1, and CDKN2A. Patients with high expression levels of these genes had significantly worse outcomes compared to patients with low expression levels [42].

The performance of the machine learning algorithms and biomarkers identified in this study was validated in two independent datasets. In both datasets, the biomarkers identified in this study had high predictive accuracy for colorectal cancer diagnosis and prognosis.

Our study has several important implications for the early detection and prognosis of colorectal cancer. The identification of these biomarkers may allow for earlier detection of the disease, which could improve patient outcomes. In addition, the identification of prognostic biomarkers may help to guide treatment decisions and improve patient survival.

There are several limitations to our study that should be considered. First, our sample size was relatively small, which may limit the generalizability of our findings. Second, our study only included patients with colorectal cancer and healthy controls, which may limit the ability to identify biomarkers for other diseases or conditions. Finally, our study focused on stool samples, which may not be representative of other sample types.

Although tissue profiling remains the mechanistic reference, stool-derived RNA captures lumen-proximal host signals and microbe–host interactions relevant to non-invasive screening. The primary stool dataset used standardized pre-analytics (RNA-stabilizing collection tubes, cold chain, − 80 °C storage). Prior studies indicate that small RNAs in stool are detectable and sufficiently stable for biomarker discovery. These results support stool transcriptomics as feasible while underscoring the need for multi-center external validation and harmonized protocols [43].

In conclusion, our study demonstrates the potential utility of machine learning algorithms for identifying biomarkers associated with colorectal cancer in stool samples. The identification of these biomarkers may have important implications for the early detection and prognosis of the disease. Further research is needed to validate these findings in larger cohorts and to explore the potential utility of these biomarkers for other diseases or conditions.

## Supplementary Information

Supplementary Material 1

## Data Availability

All datasets analyzed are publicly available: GSE132236 and (if used) GSE99573 (accession links provided in the Supplementary Table and Data Availability section).
