# Supplementary material for: Examining the gene network and prognostic biomarkers in the onset of colorectal cancer in stool samples using machine learning
Source: Discov Oncol. 2026 Mar 16;17:627. doi: 10.1007/s12672-026-04773-z (PMC13103136; doi:10.1007/s12672-026-04773-z)
Supplement: Supplementary file 1 — Supplementary Material 1 [file 12672_2026_4773_MOESM1_ESM.docx]

**Supplementary Table S1. Public datasets and metadata used in this study**

| **Accession (GEO)** | **Study (short title)** | **Citation / DOI** | **Platform** | **Data type** | **Sample type** | **N (total)** | **Group breakdown** | **Preprocessing level provided** | **Clinical labels available** | **Batch variables (inferred/annotated)** | **Used in this study (Train/Test/External)** | **Notes** |
| --- | --- | --- | --- | --- | --- | --- | --- | --- | --- | --- | --- | --- |
| GSE99573 | Clinical Performance of a Stool RNA Assay for Early Detection of Precancerous Adenomas and CRC | Barnell et al., bioRxiv 534412 (2019) | Affymetrix Human Transcriptome Array 2.0 (GPL17586) | Microarray (stool transcriptome) | Human stool-derived RNA | 338 samples listed; 330 arrayed; Train=265 / Test=65 | Normal / Adenoma / Cancer (counts not specified on GEO main page) | Processed: RMA signal intensity; Raw CEL likely available | Disease class (normal/adenoma/cancer); other clinical metadata TBD | Array run/date; GEO series | External validation (recommended) | Design: machine-learning split; suitable for external testing once harmonized |
| GSE132236 | Altered Fecal Small RNA Profiles in Colorectal Cancer Reflect Gut Microbiome Composition | Tarallo et al., mSystems 2019; DOI:10.1128/mSystems.00289-19 | Small RNA-Seq (stool); shotgun WMS (study-level) | Small RNA sequencing (human & microbial sRNAs) | Human stool (CRC, adenoma, healthy) | 80 stool specimens | CRC / Adenoma / Healthy (exact counts, see paper) | Raw FASTQ (sRNA) deposited; processed counts likely available | Disease class; limited additional metadata | Sequencing run/lane; collection site | Discovery / Feature exploration (recommended) | Integrative with metagenomics; can benchmark sRNA-based signatures |
